# Tranexamsäure zur Blutungsprophylaxe bei Trauma und orthopädischen Eingriffen – Standard oder individualisierte Anwendung?

**DOI:** 10.1007/s00101-021-00928-5

**Published:** 2021-02-23

**Authors:** Isabell Pekrul, Thomas Schachtner, Bernhard Zwißler, Patrick Möhnle

**Affiliations:** 1 Klinik für Anaesthesiologie, Abteilung für Transfusionsmedizin, Zelltherapeutika und Hämostaseologie, Universität München (LMU), Marchioninistr. 15, 81377 München, Deutschland; 2 Anästhesie und Intensivmedizin, Schön Klinik München Harlaching, München, Deutschland; 3 Klinik für Anaesthesiologie, Universität München (LMU), München, Deutschland

**Keywords:** Antifibrinolytika, Perioperativ, Off-label-use, Aufklärung, Thromboserisiko, Antifibrinolytics, Perioperative, Off-label-use, Aufklärung, Thromboembolic risk

## Abstract

Tranexamsäure hat einen etablierten Stellenwert in der Behandlung von Blutungen, v. a. bei Hyperfibrinolyse. Zunehmend wird TXA auch prophylaktisch bei Trauma und orthopädischen Eingriffen eingesetzt, wobei sich Fragen nach Risiken sowie einem möglichen Off-Label-Einsatz ergeben. Auf Basis der verfügbaren Literatur lässt sich schließen, dass ein prophylaktischer Einsatz des Präparates in diesen Indikationsbereichen vertretbar ist. Jedoch sollte bei Patienten mit erhöhtem Risiko für thrombembolische Ereignisse weiterhin eine individuelle Abwägung von Nutzen und Risiken durchgeführt und dokumentiert werden. Obwohl die Indikationsgebiete des prophylaktischen Einsatzes bei Trauma und orthopädischen Eingriffen nicht spezifisch in der Fachinformation aufgeführt sind, ist die Anwendung unseres Erachtens nicht als Off Label Use zu bewerten.

## Abstract (en)

The use of tranexamic acid (TXA) is established in the treatment of bleeding, especially of bleeding due to hyperfibrinolysis. In recent years the prophylactic use of TXA in trauma and orthopedic surgery has increased leading to open questions regarding potentially associated risks and a possible classification as off label use. The available literature provides a sound basis for the recommendation that TXA can be used in these indications provided that an individual risk assessment is done in patients with increased risks for thromboembolic complications. Although the prophylactic use of TXA in orthopedic surgery and trauma is not explicitly listed in the product characteristics, it should not be regarded as an off label use.

Seit Langem ist der Einsatz von Tranexamsäure bei der Therapie von Blutungen, vorrangig bei Hyperfibrinolyse, sowie zur Prophylaxe von Blutungen in definierten Indikationsbereichen wie kardiochirurgischen Eingriffen etabliert. In den letzten Jahren hat der Gebrauch von Tranexamsäure zur Blutungsprophylaxe als Behandlungsstandard in breiten Patientenkollektiven deutlich zugenommen. Vor allem bei Trauma sowie bei orthopädischen Eingriffen erfolgt zunehmend ein prophylaktischer Einsatz als Teil der klinischen Routine. Mit diesem Einsatzbereich des Präparates und dem damit verbundenen Wandel der Indikationen treten jedoch regelhaft folgende Fragen auf: 1) Ist ein breiter, nichtselektionierter Einsatz im Bereich der Blutungsprophylaxe mit einem Anstieg von Nebenwirkungen verbunden und 2) handelt es sich beim prophylaktischen Einsatz des Präparates um einen „Off Label Use“ bzw. sind beim prophylaktischen Einsatz ggf. besondere rechtliche Vorgaben z. B. im Rahmen der Aufklärung zu beachten?

## Historie und Wirkprinzip

Tranexamsäure (TXA) als Antifibrinolytikum wurde 1962 erstmals beschrieben, ursprünglich mit dem Ziel, ein Präparat zur Behandlung peripartaler Blutungen zur Verfügung stellen zu können [56]. Tranexamsäure ist strukturell der Aminosäure Lysin ähnlich. Der Wirkmechanismus von Tranexamsäure beruht auf der Bindung des Präparates an den Lysinrezeptor von Plasminogen, hierdurch wird die Aktivierung von Plasminogen durch Plasminogenaktivatoren („tissue plasminogen activator“, t‑PA) verhindert, was in der Folge die Auflösung von Gerinnseln durch Plasmin einschränkt [33]. Einer Hyperfibrinolyse wird somit pharmakologisch entgegengewirkt; bei aufrechterhaltener Balance zwischen Gerinnselbildung und Lyse trägt TXA durch Einschränkung der Fibrinolyse zu einer Stabilisierung des Gerinnsels bei. Die Effektstärke bei der Behandlung von Blutungen ist abhängig von der klinischen Situation; bei leichten Blutungen ist ggf. eine alleinige Therapie mit TXA ausreichend, bei schweren Blutungskomplikationen ist die Therapie mit TXA in der Regel als begleitend zur Substitution zellulärer und plasmatischer Produkte zu betrachten.

Eine intravenöse, perorale sowie topische Gabe ist möglich; aufgrund der vorwiegend renalen Ausscheidung ist eine Dosisreduktion bei schwerer Niereninsuffizienz zu beachten; das Nebenwirkungsprofil ist insgesamt günstig; gastrointestinale Beschwerden können (v. a. bei oraler Anwendung) häufiger (≥1/100 bis < 1/10) auftreten; in unbekannter Häufigkeit sind neben allergischen Reaktionen auch Sehstörungen und Kreislaufreaktionen beschrieben. Eine potenziell schwerwiegende Nebenwirkung des Präparates, das Auftreten zerebraler Krampfanfälle, ist im Wesentlichen dosisabhängig und v. a. bei Anwendung in hoher Dosierung in der Kardiochirurgie beschrieben [31]. Auf das potenzielle Risiko thrombembolischer Komplikationen wird unten detailliert eingegangen. Die Zulassung der TXA umfasst die Prophylaxe und Behandlung von Blutungen aufgrund einer generalisierten oder lokalen Hyperfibrinolyse bei Erwachsenen und Kindern ab 1 Jahr. Tranexemsäure hat einen über Jahrzehnte etablierten Stellenwert als Medikation bzw. supportive Medikation bei Menorrhagie sowie im Falle von Blutungskomplikationen bei Hämophilie, Von-Willebrand-Syndrom und weiteren seltenen Faktorenmängeln [8]. Die Anwendung bei gastrointestinaler Blutung ist nach einer aktuellen Studie, welche keinen positiven Effekt von TXA bei akuter gastrointestinaler Blutung aufzeigen konnte, umstritten [9]. Seit 2010 wurden über die lange bekannten Einsatzbereiche hinaus weitere Indikationen der TXA beschrieben und teils mit Daten großer multizentrischer klinischer Studien belegt, v. a. der Einsatz bei Trauma (CRASH-2) sowie bei peripartaler Blutung (WOMAN-Trial) [10, 58]; in beiden Indikationsbereichen ist die Substanz mittlerweile in Leitlinien und Empfehlungen aufgenommen. In den letzten Jahren wurden zudem zunehmend Studien und Empfehlungen zum prophylaktischen perioperativen Einsatz insbesondere im Rahmen von Patient-Blood-Management-Konzepten veröffentlicht.

## Tranexamsäure bei Trauma und Schädel-Hirn-Trauma

Die CRASH-2-Studie ist die bislang größte Untersuchung (multizentrisch, international, 20.211 Patienten) in dieser Indikation; sie zeigte einen Vorteil einer frühen Gabe (innerhalb 3 h nach Trauma) in Bezug auf das Überleben bei starkem Blutverlust oder Gefahr schwerer Blutungen („number needed to treat“ in Bezug auf Letalität aufgrund Blutung: 119), jedoch auch einen nachteiligen Effekt bei zu später Verabreichung (>3 h nach Trauma) [10]. Es wurde keine erhöhte Rate an arteriellen oder venösen thrombembolischen Ereignissen (ATE/VTE) in der mit TXA behandelten Gruppe beobachtet [10]. Einschränkend bei der Bewertung des Risikos thrombembolischer Ereignisse in der CRASH-2-Studie ist jedoch die Tatsache, dass solche Ereignisse zwar per Studienprotokoll registriert wurden, die Patienten aber nicht standardisiert in Bezug auf diese Nebenwirkung untersucht wurden („recorded only when clear clinical evidence“) und somit ein „underreporting“ thrombembolischer Komplikationen nicht auszuschließen ist [10]. Interessanterweise zeigte sich kein Unterschied in der Transfusionshäufigkeit zwischen der mit TXA und der ohne TXA behandelten Patientengruppe [45]. Trotz Kritik an methodischen Schwächen der Studie [4, 38] sowie Hinweisen auf eine erhöhte Inzidenz von VTE in anderen Studien [22, 37, 57] etablierte sich die Dosierung von 1 g TXA als i.v.-Bolus und einer kontinuierlichen Gabe von 1 g i.v. über 8 h in verschiedenen Leitlinien und Empfehlungen [6, 39, 43, 51] im Sinne einer frühen bzw. im engeren Sinne prophylaktischen Gabe bei schwerem Trauma.

Tranexamsäure soll in dieser Indikation einer überschießenden Hyperfibrinolyse entgegenwirken: Dieses Therapieprinzip bei Trauma ist jedoch nicht unumstritten. Kritiker argumentieren, dass die Ausprägung einer systemischen Hyperfibrinolyse von einem „fibrinolytic shutdown“, der durch das Gewebstrauma induziert wird, klinisch auch anhand von Scores und Verletzungsschwere nicht zu unterscheiden ist und eine präemptive TXA-Gabe bei Patienten mit „fibrinolytic shutdown“ zu einer Erhöhung des Risikos für thrombembolische Komplikationen und Multiorganversagen führen könnte [35]. Um dieses Risiko zu minimieren, wurde in kleineren Studien der Versuch unternommen, TXA nach viskoelastischen Messverfahren (VEM) gesteuert zu verabreichen, jedoch fand sich nur eine schwache Korrelation zwischen VEM und der nach „Goldstandard“ mittels PAP-Komplexen gemessener Hyperfibrinolyse [15, 42]. Interessanterweise zeigten jedoch eine weitere prospektive Studie sowie eine Propensity-Score-gematchte Studie, die beide bei Aufnahme im Schockraum die Auswirkung einer frühen TXA-Gabe am Unfallort mittels VEM untersuchten, eine höhere Gerinnselstabilität und einen geringeren D‑Dimer-Anstieg mit TXA; dies unterstreicht den potenziellen Stellenwert einer prophylaktischen Gabe bei Traumapatienten [28, 52].

Die CRASH-3-Studie („international, multicenter, randomised cotrolled trial“ (RCT), *n* = 12.797) fokussierte auf Patienten mit isoliertem Schädel-Hirn-Trauma (SHT). Sie erbrachte den Nachweis einer geringeren Früh- und Gesamtletalität bei Patienten mit leichtem bis mittelgradigem SHT und TXA-Gabe beim Einsatz entsprechend dem Dosisregime der CRASH-2-Studie. Dabei gab es im Vergleich zur Placebogruppe keinen Unterschied im neurologischen Outcome (Glasgow Outcome Scale), in der Häufigkeit von Krampfanfällen sowie in der Anzahl von VTE-Komplikationen [11]. Ebenso konnte eine aktuelle Metaanalyse Vorteile für den frühzeitigen Einsatz (<3 h) von TXA hinsichtlich einer geringeren Letalität und einer geringeren intrazerebralen Hämatomausbreitung bei gleicher Häufigkeit an neurochirurgischen Interventionen zeigen [23].

Zusammenfassend besteht in Leitlinien Konsens für eine frühzeitige Gabe von TXA bei Traumapatienten; Ergebnisse von weiteren Studien zur idealen Dosierung und zum optimalen Zeitpunkt der Gabe sind noch ausstehend. Eine vorab durchgeführte VEM-Testung wird nicht empfohlen [51]. Angesichts einer möglicherweise erhöhten VTE-Inzidenz unter TXA ist jedoch der Stellenwert einer VTE-Prophylaxe mit physikalischen und ggf. medikamentösen Maßnahmen im späteren Verlauf zu betonen [2].

## Prophylaktische Anwendung in der Orthopädie

In den letzten Jahren wird TXA v. a. bei elektiven endoprothetischen Knie- und Hüftgelenkoperationen (KTEP/HTEP) auch zunehmend als Prophylaxe zur Reduktion von Blutungen eingesetzt. Allerdings wird die TXA-Applikation in diesem Anwendungsbereich sowohl innerhalb der Fachdisziplinen als auch zwischen anästhesiologischer und orthopädischer Fachdisziplin durchaus kontrovers diskutiert. Befürworter einer prophylaktischen TXA-Gabe führen als Argumente reduzierte Blutungsvolumina sowie eine reduzierte Transfusionswahrscheinlichkeit, aber auch eine verkürzte Erholungszeit und reduzierte Inflammation ins Feld; Kritiker argumentieren mit einem potenziell erhöhten Risiko für thrombembolische Komplikationen. Durch die zunehmende Fülle und Heterogenität an Studien und Metaanalysen der letzten Jahre im Hinblick auf unterschiedliche Einschlusskriterien, Dosisregime, Applikationsrouten und Endpunkte erscheint eine Konsensfindung erschwert.

### Effektstärke

Als primäre Outcome-Parameter werden in den kontrollierten Studien meist die Reduktion der Transfusionswahrscheinlichkeit und des perioperativen Blutverlustes genannt. Die Vermeidung der allogenen Bluttransfusion stellt ein Kernelement vieler „Enhanced-recovery-after-surgery“(ERAS)*-*Programme dar; für die Fremdblutgabe wurden Assoziationen mit einem erhöhten Infektionsrisiko und einer Verlängerung der Krankenhausverweildauer als auch mit einer erhöhten Mortalität gezeigt [3, 26]. Die Inzidenz der allogenen Fremdbluttransfusion ist allerdings sehr variabel: Es werden z. B. Inzidenzen von 2,5–35,3 % für KTEP- und 14–29,8 % für HTEP-Operationen berichtet [50]. Neben operationstechnischen Unterschieden sind weitere unabhängige Risikofaktoren für eine Fremdblutgabe gut beschrieben und umfassen beispielsweise (1) die unbehandelte, präoperative Anämie, welche mit einer Prävalenz von ca. 14–23 % beobachtet wird [29, 34] sowie (2) verlängerte Operationszeiten [7, 46]. Darüber hinaus nehmen auch unterschiedliche Transfusionstrigger sowie das Flüssigkeits- und Volumenmanagement einen Einfluss. Die Messung bzw. Schätzung des Blutverlustes stellt ebenso eine Herausforderung dar und unterliegt verschiedenen Limitationen, die meist zu einer Überschätzung des Blutverlustes führen [20]. Die Variabilität der Effektstärke zwischen einzelnen Studien ist hierdurch erklärbar. Trotz dieser hohen Variabilität konnte der blutsparende Effekt der prophylaktischen TXA-Gabe jedoch bei verschiedenen chirurgischen Eingriffen, in verschiedenen Gesundheitssystemen und Ethnien reproduziert werden. Einen Anhalt zur Abschätzung der Effektstärke bei operativen Eingriffen in unterschiedlichen Disziplinen bietet die Metaanalyse von Ker et al., die eine Reduktion des Blutverlustes und der Transfusion bis zu einem Drittel nach TXA-Gabe aufzeigen konnte (Tab. 1; [25]).

**Table 1:** 

Art der Operation	Anzahl der Ereignisse (Tranexamsäure/Kontrolle)	Gepooltes Risiko-Verhältnis (95 % KI)	*p-*Wert^a^	Heterogenität	
Herzchirurgie	622/835	0,65 (0,60–0,70)	<0,001	60	<0,001
Orthopädische Operationen	298/462	0,55 (0,49–0,61)	<0,001	83	<0,001
Leberchirurgie	29/54	0,52 (0,39–0,68)	<0,001	93	<0,001
Urologische Operationen	40/60	0,66 (0,48–0,91)	0,01	2	0,31
Gefäßchirurgie	11/19	0,58 (0,34–0,99)	0,05	–	–
Gynäkologische Operationen	17/50	0,86 (0,48–1,54)	0,61	65	0,06
Schädel‐ und Kieferchirurgie	52/76	0,63 (0,45–0,86)	0,004	46	0,12

^a^Test auf Effekt

Die Unterstützung der Antifibrinolyse mittels TXA vermag durch die Verbesserung der Gerinnselstabilität die prokoagulatorische Seite des Blutgerinnungssystems für den Zeitraum der Operation zu unterstützen; speziell bei HTEP-Operationen gibt es Hinweise auf eine mögliche Induktion einer lokalen Fibrinolyse durch das operative Trauma [5, 13]. Für den Anwendungsbereich „Endoprothesenchirurgie“ wird die blutsparende Wirkung der TXA-Applikation in Metaanalysen in relevanter Größenordnung beschrieben [1, 17, 18, 36, 53, 62] z. B. eine signifikante Reduktion der Transfusionsrate auf 8,2 % (vs. 19,5 % bei Placebo) und des Blutverlustes von 1089,6 ± 1251,5 ml (vs. 1410,3 ± 1111,0 ml bei Placebo) [36]. Auf Basis dieser Daten sprechen sich zwei Fachgesellschaften für die Anwendung von TXA bei KTEP- und HTEP-Operationen aus [16, 47].

### Dosierung und Applikationsweg

Trotz des Konsenses über den Vorteil einer perioperativen TXA-Gabe in Leitlinien und Empfehlungen bestehen Differenzen zu Dosierung und Applikation. Die deutsche Handlungsempfehlung der Arbeitsgemeinschaft Endoprothetik (AE) gibt eine konkrete Dosierempfehlung: (1) die einmalige präoperative i.v.-Gabe von 15 mg/kgKG oder (2) 2 g oral 2 h vor Abruf in den OP und/oder die topische Applikation von weiteren 1–2 g Tranexamsäure. Die amerikanische Fachgesellschaft der Knie- und Hüftchirurgen (AAHKS) hingegen gibt z. B. keine spezifische Dosisempfehlung, empfiehlt aber eine Einmaldosis unabhängig vom Applikationsweg; sofern eine i.v.-Applikation vorgesehen ist, sollte diese präoperativ erfolgen [16, 47].

### Sicherheit

KTEP- und HTEP-Operationen sind mit einem Risiko für thrombembolische Komplikationen verbunden. Es besteht die Sorge, dass die antifibrinolytische Wirkung von TXA neben anderen bekannten transienten Risikofaktoren wie (1) Operationsdauer, (2) operationstechnischen Aspekten (z. B. Anwendung von Tourniquets), (3) Akute-Phase-Reaktion oder (4) längere Immobilisierungsphasen einen zusätzlichen risikoerhöhenden Effekt auf die Auftretenswahrscheinlichkeit von VTE ausüben könnte [24, 61]. Während nach früheren Daten die Inzidenz der tiefen Beinvenenthrombose (TVT) bei Endoprothesenoperationen mit bis zu 60 % beobachtet wurde, sinkt über die letzten Jahrzehnte das prozedurbezogene TVT-Basisrisiko [2]. Dieser Effekt ist einerseits auf den standardisierten Einsatz der medikamentösen und physikalischen Thromboseprophylaxe zurückzuführen, andererseits führt die Ausrichtung aller orthopädischen und anästhesiologischen Prozesse auf die Umsetzbarkeit einer stringenten Frühmobilisierung innerhalb von 24 h nach der Operation zur weiteren Senkung der TVT-Rate (z. B. 0,4 % in einer prospektiven Observationsstudie an 17.582 Eingriffen) [14, 21, 40, 48, 49]. Hierzu kann jedoch auch die prophylaktische TXA durch eine Verminderung der lokalen Schwellung und Erleichterung der Frühmobilisierung und Rehabilitation beitragen [12, 27, 44]. Ergebnisse von Metaanalysen zeigen überwiegend keine signifikante Erhöhung des TVT-Risikos nach TXA-Gabe bei endoprothetischen Eingriffen [18, 53–55, 60]. Diese Aussage ist jedoch mit Limitationen behaftet, aufgrund der Heterogenität der Studienprotokolle, der statistischen Unterpowerung der RCT hinsichtlich dieses Ereignisses und eines möglichen Publikationsbias. Schließlich sind auch durchaus gegenteilige Ergebnisse publiziert, die eine erhöhte Rate an VTE in der TXA-Gruppe (vs. Placebo; 4,04 vs. 1,71 %) beschreiben [36]: In diese Metaanalyse von Moskal et al. wurde jedoch eine Studie eingeschlossen, welche sowohl im Placeboarm als auch in den 4 Therapiearmen eine ungewöhnlich hohe TVT-Rate von 9 bis 13 % aufwies [19]. Auf Basis aller vorliegenden Daten fällt die Risiko-Nutzen-Bewertung jedoch insgesamt günstig für die Anwendung von TXA aus, sofern die Patienten kein erhöhtes Risiko für ATE und/oder VTE aufweisen.

Die prophylaktische TXA-Gabe wird neben der Endoprothetik zunehmend auch bei anderen großen orthopädischen Eingriffen mit relevantem Blutungsrisiko eingesetzt. Bei Wirbelsäuleneingriffen wurde eine Reduktion des perioperativen Blutverlustes und Transfusionsrisikos (relatives Risiko 0,65; 95 %-Konfidenzintervall; 0,53–0,85) gezeigt [32, 59]. In der Mehrzahl der Studien wird angesichts längerer Operationszeiten im Vergleich zur Endoprothesenchirurgie nach der präoperativen Bolusgabe von 10–30 mg/kgKG eine Erhaltungsdosis von 1–2 mg/kgKG und h gegeben [32]. Bewertet man andererseits das VTE-Risiko bei diesen Eingriffen, sollten die oftmals längeren Operationszeiten, spezielle Lagerungstechniken sowie Einschränkungen der postoperativen Mobilisierbarkeit als potenzielle Risikofaktoren berücksichtigt werden.

### Einsatz bei erhöhtem Risiko für thrombembolische Komplikationen

Gemäß Fachinformation sind thrombembolische Erkrankungen und/oder Risikofaktoren für thrombembolische Ereignisse Kontraindikationen für die Anwendung von TXA. Bei thrombophilen Hochrisikopatienten darf gemäß Fachinformation *„Tranexamsäure nur bei strenger Indikationsstellung nach Rücksprache mit einem hämostaseologisch erfahrenen Arzt und unter engmaschiger medizinischer Überwachung angewendet werden.“ *Dies ist v. a. bei einer evtl. Anwendung innerhalb von perioperativen Standards zu bedenken, da dies auch eine Anamnese für thrombembolische Ereignisse voraussetzt, welche entsprechend dokumentiert vorliegen sollte.

Eine aktuelle Metaanalyse fokussiert auf Risikopatienten und untersucht hierzu die Inzidenz von VTE in risikoselektionierten vs. nichtrisikoselektionierten Patientenpopulationen: Die Ergebnisse zeigen zwar keine erhöhte Inzidenz für VTE in den Studiengruppen (TXA systemisch vs. Placebo, RR 0,95 (0,78–1,15)), die Autoren verweisen aber auf eine aktuell nichtausreichende Datenlage, um eine Anwendungsempfehlung in diesem Kollektiv geben zu können [60]. Die amerikanischen Fachgesellschaften raten in der praktischen Umsetzung zu einer individualisierten Risiko-Nutzen-Bewertung [16]. Die deutsche Arbeitsgemeinschaft für Endoprothetik empfiehlt, bei Hochrisikopatienten die Gabe von TXA zu vermeiden [47].

Bei Patienten mit Risikofaktoren für thrombembolische Ereignisse ist aus Sicht der Autoren eine Gabe von TXA nicht ausgeschlossen, es ist jedoch eine individuelle Abwägung von Risiko und dem potenziellen Nutzen einer prophylaktischen Gabe dieses Antifibrinolytikums notwendig [30], welche zudem sorgfältig dokumentiert werden sollte. Die Balance von Hämostase und Thromboseneigung unterliegt einer Vielzahl an Einflussfaktoren, welche sich zwischen einzelnen Kliniken deutlich unterscheiden: z. B. durch Unterschiede in Operationstechnik und -dauer, Lagerung, Beginn der Mobilisation, Standard zur medikamentösen Thromboseprophylaxe. Aus diesem Grund sind allgemeingültige Empfehlungen für den Einsatz von TXA bei diesen Risikopatienten nicht sinnvoll; hier muss auf der Basis von institutionellen Bedingungen wie auch patientenbezogenen Risikofaktoren individuell entschieden werden.

## Prophylaktische Gabe von Tranexamsäure – ein „Off Label Use?“

Tranexamsäure ist indiziert zu „Prophylaxe und Behandlung von Blutungen aufgrund einer lokalen oder generalisierten Hyperfibrinolyse“ (Zitat Fachinformation „Cyklokapron®-Injektionslösung, Fa. Pfizer“ [41]), zudem werden in der Fachinformation Hals‑, Nasen‑, Ohrenoperationen, gynäkologische Operationen, geburtshilfliche Blutungen und Operationen in Thorax und Bauchraum sowie andere größere chirurgische Eingriffe wie z. B. kardiovaskuläre Operationen aufgeführt. Der Einsatz bei Traumapatienten zur Prophylaxe einer durch Hyperfibrinolyse bedingten Blutung ist aus Sicht der Autoren über diese Zulassung abgedeckt. Der Einsatz zur Prophylaxe von Blutungen bei größeren operativen Eingriffen erscheint gerechtfertigt, auch wenn einzelne operative Verfahren wie z. B. Endoprothetik nicht explizit als Indikationsbereich in der Fachinformation aufgeführt sind.

Zum Begriff der „lokalen Hyperfibrinolyse“ in der Fachinformation ist kritisch anzumerken, dass der Nachweis einer Hyperfibrinolyse, insbesondere wenn sie auf den Ort der Verletzung oder das Operationsgebiet beschränkt ist, in der klinischen Routine weder vorgesehen noch einfach durchzuführen ist, dass jedoch eine auch lokalisierte Hyperfibrinolyse bei oben genannten Operationen durchaus auftreten kann [5, 13]; jedoch ist aus Sicht der Autoren bei operativen Eingriffen der Nachweis einer Hyperfibrinolyse generell keine zwingende Vorbedingung für den Einsatz des Präparates innerhalb der Zulassung, da dies über den Passus „Prophylaxe“ abgedeckt ist.

Herr Dr. jur. Elmar Biermann (Justiziar der Deutschen Gesellschaft für Anästhesiologie und Intensivmedizin) bewertet die Fragestellung wie folgt (persönliche Kommunikation an Prof. Zwißler vom 30.07.2019):

Gemäß Fachinformation ist Tranexamsäure indiziert zur Prophylaxe und zur Behandlung von Blutungen aufgrund einer lokalen oder generalisierten Hyperfibrinolyse. Im Folgenden gibt die Fachinformation dann die „genauen“ Anwendungsgebiete an. An erster Stelle werden wieder Blutungen aufgrund einer lokalen und generalisierten Hyperfibrinolyse genannt, dann werden beispielhaft drei „Blutungssituationen“ umschrieben. In den nächsten Spiegelstrichen werden bestimmte Eingriffe genannt, der vorletzte Spiegelstrich betrifft Operationen an Thorax und Bauchraum sowie andere größere chirurgische Eingriffe wie z. B. kardiovaskuläre Operationen. Der Zusatz „wie z. B.“ öffnet den Anwendungsbereich der Tranexamsäure über die beschriebenen Eingriffe hinaus. Hätte die Fachinformation andere größere Operationen ausschließen wollen, dann hätte sie die Beispiele als „ausschließlich“ einleiten oder sonstige Eingriffe als kontraindiziert bezeichnen müssen. Die häufige Verwendung des Hinweises „z. B.“ macht deutlich, dass es zwar um Blutungen aufgrund lokaler oder generalisierter Hyperfibrinolyse geht, dass die Operationen aber gerade nicht abschließend dargestellt sind. Im Ergebnis sehe ich deshalb nicht, dass die Behandlung von Blutungen aufgrund lokaler oder generalisierter Hyperfibrinolyse bei orthopädischen Eingriffen eine Behandlung außerhalb des vom Hersteller definierten Anwendungsgebietes wäre. Insofern stellt sich meines Erachtens die Aufklärungsproblematik unter dem speziellen Aspekt „Off Label Use“ nicht.

Eine dezidierte Aufklärung über einen Off Label Use wird somit aus Sicht der Autoren bei prophylaktischer Gabe in den oben aufgeführten Indikationen in der Regel nicht benötigt, die Indikation zur Gabe sollte jedoch dokumentiert sein. Eine wesentliche Bedingung für dieses Vorgehen ist jedoch, dass das Präparat nicht bei bekannten Kontraindikationen bzw. nur nach sorgfältiger, individueller Risiko-Nutzen-Abwägung eingesetzt wird.

## Fazit für die Praxis

- Tranexamsäure kann in der Prophylaxe von Blutungen bei Trauma und orthopädischen Eingriffen eingesetzt werden.
- Bei Patienten mit thrombembolischen Risikofaktoren sollte eine individuelle Nutzen-Risiko-Abwägung unter Berücksichtigung klinikspezifischer Rahmenbedingungen, wie z. B. Operationsstandards und Standards bei der Mobilisation und Thromboseprophylaxe, erfolgen.
- Eine Anwendung von Tranexamsäure in der Prophylaxe von Blutungen bei Trauma und orthopädischen Eingriffen ist nicht als „Off Label Use“ zu werten.
